# Early start of the West Nile fever transmission season 2018 in Europe

**DOI:** 10.2807/1560-7917.ES.2018.23.32.1800428

**Published:** 2018-08-09

**Authors:** Joana M. Haussig, Johanna J. Young, Céline M. Gossner, Eszter Mezei, Antonino Bella, Anca Sirbu, Danai Pervanidou, Mitra B. Drakulovic, Bertrand Sudre

**Affiliations:** 1 European Centre for Disease Prevention and Control (ECDC), Solna, Sweden; 2 These authors contributed equally to this article and share first authorship; 3 Ministry of Human Capacities, Budapest, Hungary; 4 Department of Infectious Diseases, Istituto Superiore di Sanità (ISS), Rome, Italy; 5 National Institute of Public Health, Bucharest, Romania; 6 Hellenic Center for Disease Control & Prevention, Marousi, Greece; 7 National Institute of Public Health “Dr Milan Jovanovic-Batut”, Belgrade, Serbia

**Keywords:** West Nile fever, West Nile virus, real-time surveillance, vector-borne diseases

## Abstract

In Europe, surveillance indicates that the 2018 West Nile fever transmission season started earlier than in previous years and with a steeper increase of locally-acquired human infections. Between 2014 and 2017, European Union/European Economic Area (EU/EEA) and EU enlargement countries notified five to 25 cases in weeks 25 to 31 compared with 168 cases in 2018. Clinicians and public health authorities should be alerted to ensure timely implementation of prevention measures including blood safety measures.

During the West Nile fever (WNF) transmission season in Europe lasting from June to November, European Union/European Economic Area (EU/EEA) and EU enlargement countries monitor the occurrence of all probable and confirmed human WNF cases [1]. The observed occurrence of an unusually early start of the transmission season and the increase in the number of locally-acquired human cases this year prompted us to investigate this signal (excluding asymptomatic cases among blood donors) in comparison with previous seasons and assess the public health implications.

## Description of the start of the West Nile fever season 2018

**Figure 1:**
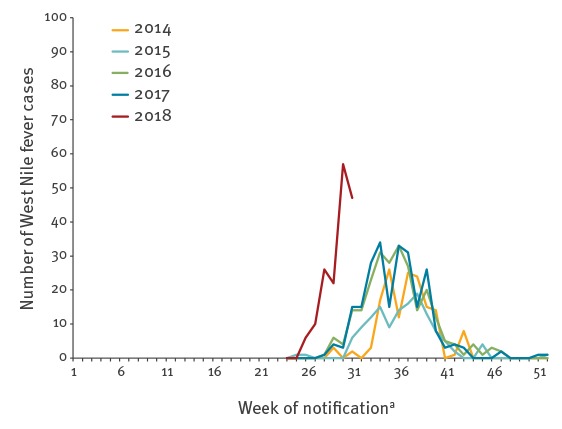
Number of West Nile fever cases in European Union/European Economic Area (EU/EEA) and EU enlargement countries by epidemiological week of notification^a^, 2014–2018 (n = 943)

^a^ Week of notification to national authorities or if missing, week of notification to the European Centre for Disease Prevention and Control (ECDC).

Between 2014 and 2018, EU/EEA and EU enlargement countries (Austria, Bulgaria, Croatia, Cyprus, France, Greece, Hungary, Italy, Kosovo*, Portugal, Romania, Spain, Serbia and Turkey) reported locally-acquired WNF cases. WNF follows a seasonal pattern with most cases acquired in the countries above reported between July and October, with the case numbers usually peaking between mid-August and mid-September (Figure 1). In the previous two years (2016 and 2017), the first cases during the WNF transmission season were notified from week 28 onwards (Figure 1).

In 2018, the first WNF cases were notified by Greece in week 26 (25 June to 1 July), with the earliest disease onset on 31 May (week 22). Additionally, we observed an apparent steeper increase of reported case numbers in this season compared with previous years (Figure 1). Between 2014 and 2017, five to 25 cases were notified from week 25 to week 31, in marked contrast to 168 cases reported in the same time period in 2018 by EU/EEA and EU enlargement countries.

Compared to the six-week period after the initial case report for each year since 2014, the number of cases reported in 2018 is above previous years, with 168 cases in 2018 in comparison with eight in 2014, two in 2015, 62 in 2016 and 66 in 2017.

In 2018, the yearly median age of cases is 66 years and the sex ratio (male: female) is 2.1 among cases reported to date. Both values are comparable to those in the previous four years (median age between 64.5 and 68 years and sex ratio between 1.3 and 2.4).

As in previous years, Greece has reported the identification of West Nile virus lineage 2 (WNV-2) in 2018, which has been identified regularly throughout south, east and central Europe since its first detection in 2008 in Hungary [2].

Data reported to the Animal Disease Notification System (ADNS) of the European Commission also indicate an early start of the transmission season in 2018 among equids, with six outbreaks among equids between week 26 and week 31 reported by Hungary (3 outbreaks), Greece (2 outbreak) and Italy (1 outbreak). In comparison, one to two outbreaks were reported in the same time period between 2014 to 2016. In 2017, 10 outbreaks were reported in the same period by Italy and Greece.

**Figure 2:**
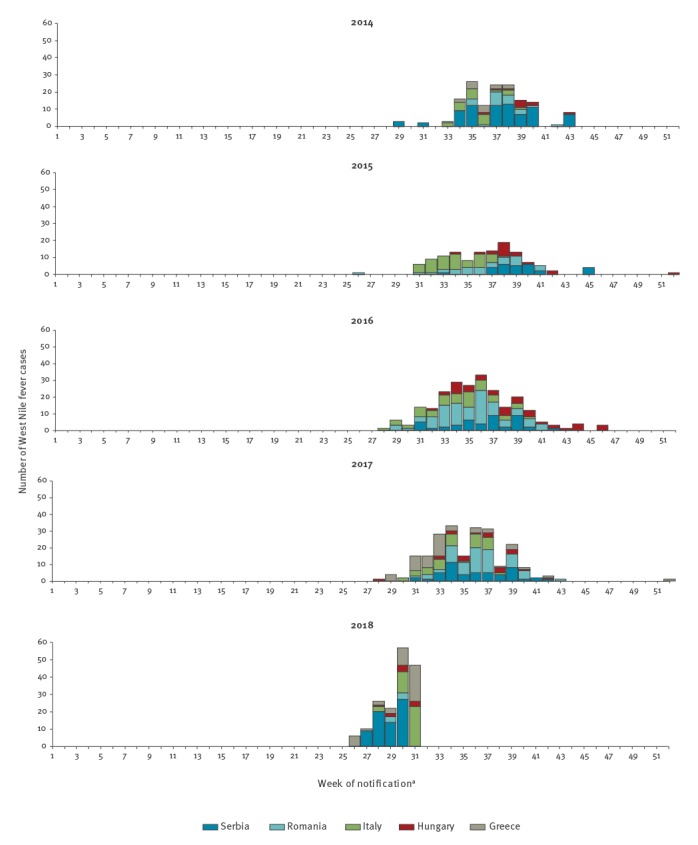
Number of West Nile fever cases by week of notification^a^ and by year, for Greece, Hungary, Italy, Romania and Serbia, 2014–2018 (n = 899)

^a^ Week of notification to national authorities or if missing, week of notification to the European Centre for Disease Prevention and Control (ECDC).

Since the beginning of 2018 and as at 3 August, among the 168 human cases reported, 98 have been reported by EU/EEA countries including Greece (43 cases), Italy (38), Hungary (10), and Romania (7). Italy reported two deaths. Furthermore, in the EU enlargement countries 70 human cases, including four deaths, have been reported, all by Serbia (Figure 2).

**Figure 3:**
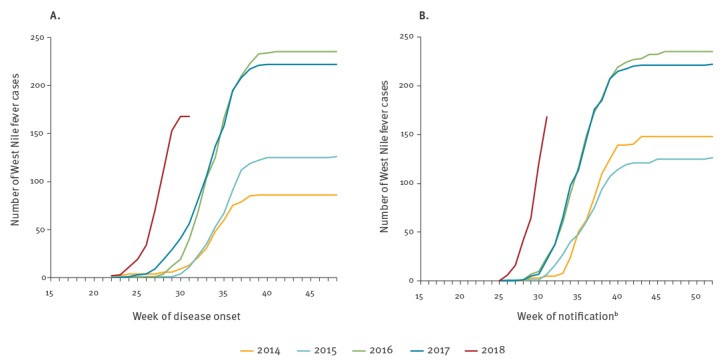
Cumulative number of cases by week of disease onset (n = 837^a^) (A) and by week of notification (n = 899) (B), Greece, Hungary, Italy, Romania and Serbia, 2014–2018

^a^ Of 899 cases notified during 2014–2018, 62 cases were missing information on the week of disease onset for 2014.

^b^ In panel B week of notification to national authorities or if missing, week of notification to the European Centre for Disease Prevention and Control (ECDC).

Real-time WNF surveillance is highly dependent on timely reporting of cases and completeness of data such as the date of onset. To verify that the higher number of cases earlier in the season is not caused by faster reporting, or biased by missing weeks of onset (62 missing weeks of onset for 2014), we analysed cumulative case numbers by week of disease onset (Figure 3A), as well as by week of reporting to the national level, or if missing, by week of notification to the European Centre for Disease Prevention and Control (ECDC) (Figure 3B). Both analyses show a similar pattern, with the only difference being that for analyses by week of onset the cumulative endpoint of number of cases is lower due to exclusion of cases with missing weeks of onset. This is an indication that the current WNF season truly started earlier and this cannot be explained by faster reporting.

## Geographical distribution

**Figure 4:**
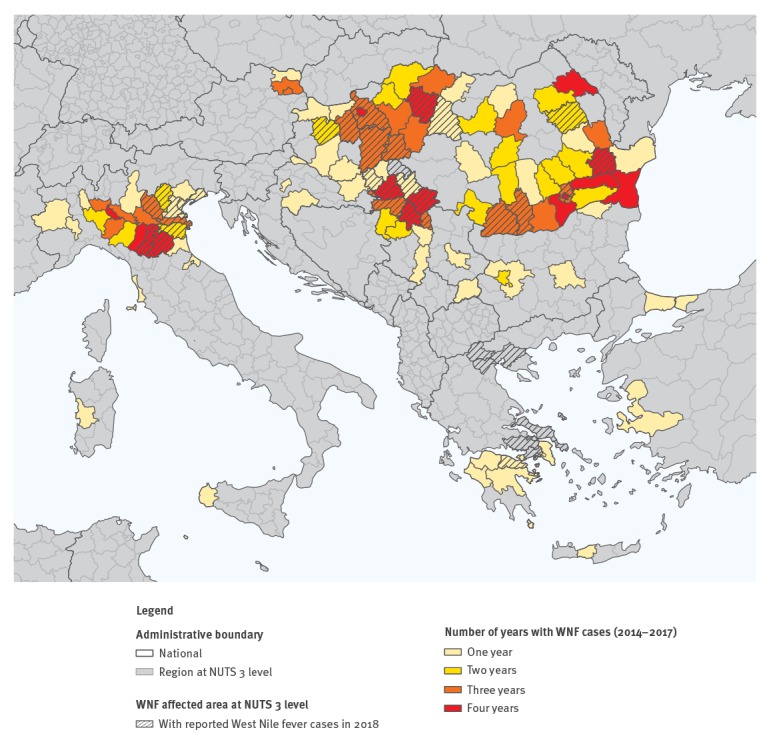
Distribution of regions at NUTS 3 level reporting at least one case of West Nile fever, Europe, 2014–2018

NUTS: Nomenclature of Territorial Units for Statistics; WNF: West Nile fever.

So far, the majority of the cases in 2018 were reported from previously affected regions at Nomenclature of Territorial Units for Statistics (NUTS) 3 level between 2014 and 2017, with an exception of few areas in Greece. The early affected regions at NUTS 3 level in 2018 are mostly regions that had already reported cases at least three or four times between 2014 and 2017 (Figure 4).

## Public health implications

WNF is a vector-borne disease caused by a flavivirus that is mostly transmitted through bites of *Culex* mosquitoes. It can also be transmitted through organ transplantation, blood transfusion, from mother to foetus during pregnancy and in laboratory settings [3]. In Europe, WNV has caused both sporadic cases as well as large outbreaks of WNF among humans [4-6].

Real-time monitoring of WNF cases is instrumental to ensure that clinicians and public health authorities are informed about WNV transmission and receive early warnings about unusual seasonal patterns. This also ensures that blood safety measures are implemented in a timely manner to avoid infections through blood transfusions. According to the Commission Directive 2014/110/EU, prospective blood donors should be deferred for 28 days after leaving a risk area of locally acquired WNV unless the result of an individual nucleic acid test (NAT) is negative [7]. In order to assist national authorities in implementing these measures, including for travellers returning from risk areas, ECDC publishes weekly updated maps of human WNF cases at the NUTS 3 level during the WNF transmission season [8]. Moreover, these reports define the geographical areas for which persons residing or visiting, and especially persons at higher risk of severe WNF (i.e. the elderly and immunocompromised) should be promptly informed about the preventive measures to reduce mosquito biting such as use of mosquito repellent and adequate clothing.

According to monthly summaries of Copernicus Climate Change Services, a European system that monitors environmental and societal challenges associated with human-induced climate changes, the average precipitation observed in March 2018 was above the 1981–2010 period average in many parts of Europe, notably in the WNF affected areas [9]. In April 2018, the surface air temperatures presented a marked anomaly above average while precipitation was almost normal. Temperatures in May 2018 were also higher than the 1981–2010 average in the WNF affected areas. Of note, precipitation in Italy and the countries along the Adriatic coast was much above average in May. June 2018 saw precipitation much above the average in most of southern Europe, and in particular, in the countries along the Adriatic coast, with floods in several regions, including in Greece and Romania [9].

This observed weather pattern is indicative of an early spring season in the south-eastern part of Europe and might have sustained environmental conditions favouring an early upsurge of the vector population. This is confirmed by the recent observation from the Serbian Programme for WNV surveillance, which includes surveillance of mosquitoes, birds and horses, carried out since 2013 [10]. In 23 of 65 mosquito sampling stations (MSS), established across seven north Serbian districts, WNV-RNA was detected in *Culex pipiens* species, in week 25 (collecting time, 19–22 June), 2018. That is the highest number of positive MSS ever detected, in those districts (data not shown). This observation could be explained by high temperatures in April and May 2018, with the highest ever recorded average April temperatures since 1888 in Serbia.

Several studies have shown that certain environmental factors, such as temperature and precipitation anomalies, can be predictors for WNF transmission [11-13]. Elevated temperatures can increase virus replication and shorten the incubation period within the mosquito, which can facilitate virus circulation and therefore WNV outbreaks [14]. The underlying determinants of this early upsurge, such as specific environmental conditions in 2018, are not fully understood to date. An in-depth study of the environmental determinants would be required to assess the association of WNF and the observed climate pattern over a longer time period in Europe.

The early start of the WNF transmission season observed in 2018 should prompt early awareness raising among clinicians and public health authorities, as the observed pattern might constitute an early sign of a season with larger outbreaks if environmental conditions remain favourable for WNV transmission. The occurrence and extent of outbreaks, however, depend on numerous factors and the situation at present does not necessarily mean that a large outbreak will happen. As the first disease onset this year occurred at the end of May, the previous assumption that the WNF season and the related period of enhanced real-time monitoring of WNF in Europe is typically from mid-June to November, may have to be reconsidered.

## Note

*This designation is without prejudice to positions on status, and is in line with United Nations Security Council Resolution 1244/99 and the International Court of Justice Opinion on the Kosovo Declaration of Independence.
